# Identification of eIF6 as a prognostic factor that drives tumor progression and predicts arsenic trioxide efficacy in lung adenocarcinoma

**DOI:** 10.1007/s11033-022-07917-w

**Published:** 2022-11-26

**Authors:** Yan Shen, Ruihong Zhang, Xiangrui Li

**Affiliations:** 1 College of Veterinary Medicine, Nanjing Agricultural University, Nanjing, Jiangsu P.R. China; 2 State Key Laboratory of Medical Genomics, Research Center for Experimental Medicine, Rui-Jin Hospital, Shanghai Jiao Tong University School of Medicine, Shanghai, China; 3 Shanghai Institute of Hematology, State Key Laboratory of Medical Genomics, Ruijin Hospital, National Research Center for Translational Medicine at Shanghai, Shanghai Jiao Tong University School of Medicine, 200000 Shanghai, P.R. China

**Keywords:** Eukaryotic translation initiation factor 6, Proliferation, Stemness, Lung adenocarcinoma, Arsenic trioxide

## Abstract

**Background:**

Lung cancer is the leading cause of cancer-related mortality worldwide. Dysregulation of mRNA translation can contribute to the development and progression of cancer whilst also having an impact on the prognosis of different types of malignancies. Eukaryotic translation initiation factors (eIFs) have been reported to serve a key role in the initiation of mRNA translation. However, little was known about the association between eIF6 and lung adenocarcinoma (LUAD) progression. We aimed to elucidate the roles of eIF6 in LUAD tumorigenesis.

**Methods:**

Bioinformatic analysis was conducted to assess the clinical significance of eIF6 in LUAD. CCK-8, colony formation assays were used to evaluate the biological roles of eIF6. The subcutaneous model was used to assess the in vivo roles of eIF6.

**Results:**

In the present study, it was found that eIF6 expression was significantly higher in LUAD samples compared with that in normal lung tissues. Higher expression levels of eIF6 were found to be associated with more advanced clinical stages of LUAD and poorer prognoses in patients with LUAD. Subsequently, overexpression of eIF6 was demonstrated to promote LUAD cell proliferation, migration and invasion, which are features of metastasis, in vitro. By contrast, inhibition of eIF6 induced cell cycle arrest and apoptosis in LUAD cells. Further bioinformatics analysis and experimental assays revealed that eIF6 expression positively correlated with the mRNA expression of stemness-associated genes in LUAD cells. Targeting eIF6 suppressed the sphere formation capacity of LUAD cells. In addition, data from the subcutaneous xenograft model in vivo also suggested that eIF6 deficiency could significantly delay tumor growth and improve the prognosis of mice. Targeting eIF6 rendered LUAD cells sensitive to arsenic trioxide treatment.

**Conclusion:**

The present study suggest that eIF6 can serve as a prognostic biomarker and a potential therapeutic target for patients with LUAD.

**Supplementary Information:**

The online version contains supplementary material available at 10.1007/s11033-022-07917-w.

## Introduction

Lung cancer is an aggressive malignancy that is the leading cause of cancer-associated mortality worldwide [1]. Despite recent developments in novel treatment strategies, the prognosis for patients with lung cancer remains dismal [2]. Lung cancer can be classified into two major types, namely small-cell lung cancer and non-small cell lung cancer (NSCLC). In particular, lung adenocarcinoma (LUAD) is one of the major subtypes of NSCLC, which occurs primarily in the distal airways [3]. Elevated epidermal growth factor receptor (EGFR) kinase activity, as a result of increased expression and/or gain-of-function mutations, is largely responsible for the tumorigenesis of LUADs [4]. Due to this kinase activity of EGFR, tyrosine kinase inhibitors (TKIs), including gefitinib, afatinib and osimertinib, have been developed to target this receptor [5]. Although these EGFR inhibitors have demonstrated potent efficacy for treating LUADs harboring EGFR with activating mutations initially, a large proportion of patients with LUADs eventually develop drug resistance [6]. At present, circumventing this resistance to TKIs remains a formidable challenge in the treatment of NSCLC. Therefore, elucidating the mechanism underlying the molecular pathogenesis of LUAD is pivotal for the discovery of novel therapeutic strategies to improve prognosis.

Dysregulation of mRNA translation has been previously reported to contribute to the carcinogenic process [7–9]. In addition, this pathophysiological process has been shown to affect the therapeutic efficacy and prognosis of different types of malignancies [7–9]. Eukaryotic translation initiation factors (eIFs) serve a key role in mRNA translation initiation. Aberrant expression of eIFs has been demonstrated to be one of the causes of tumorigenesis and progression [10]. Overexpression of eIF4E has been shown to promote malignant transformation, where patients with higher eIF4E expression levels tended to have poorer prognoses. In colorectal cancer, eIF4E overexpression was shown to promote integrin β1 translation, which resulted in 5-fluorouracil chemoresistance [11]. By contrast, the protein expression levels of eIF1, eIF2D, eIF3C and eIF6 were all found to be reduced in pancreatic ductal adenocarcinoma samples compared with those in non-neoplastic pancreatic tissues [12]. Therefore, different genes of eIFs may mediate distinct roles in different types of cancers. Elucidating the precise function of eIFs can assist in the discovery of novel molecular mechanisms involved in cancer formation and progression.

eIF6 is a conserved 27-kDa protein that has dual function in eukaryotes. In the cytoplasm, eIF6 can function as an anti-association factor to prevent the assembly of the 40 and 60 S ribosomal subunits [13]. In the nucleus, eIF6 serves an essential role in ribosome biogenesis. Previous studies have revealed that eIF6 is also an important factor in liver cancer tumorigenesis and progression [14, 15]. Dysregulation of eIF6 function and expression has been previously discovered in various types of malignancies, including breast cancer, colorectal carcinoma (CRC), malignant pleural mesothelioma (MPM) and ovarian adenocarcinoma [16–18]. In CRC and MPM, eIF6 exhibited higher expression levels compared with those in corresponding non-neoplastic tissues, suggesting a potential role for eIF6 in carcinogenesis [19, 20]. In addition, eIF6 has been found to indirectly regulate Wnt/β-catenin signaling and activate a number of AKT-associated signaling pathways in CRC [16]. In ovarian cancer cells, eIF6 can affect CDC42 signaling to promote migration and invasion [21].

In the present study, the aim is to investigate the potential role of eIF6 in LUADs. It was found that the expression of eIF6 is higher in LUAD tissues compared with that in normal tissues. Higher eIF6 expression levels associated positively with tumor progression, suggesting that eIF6 can serve as a prognostic biomarker for patients with LUAD. In addition, eIF6 was found to promote LUAD cell proliferation and metastasis, whereas knocking down eIF6 expression inhibited cell proliferation and induced apoptosis of LUAD cells. Therefore, these results revealed the potentially key role of eIF6 in the development of LUADs, implicating it as a therapeutic target for improving the prognosis of LUADs.

## Materials and methods

### Cell lines and culture

Human LUAD cell lines A549, NCI-H1299 and NCI-H2122 were obtained from the Culture Collection of the Chinese Academy of Science (Shanghai, China) in 2019. All cell lines were cultured in RPMI 1640 media (Gibco; Thermo Fisher Scientific, Inc.) containing 10% FBS (Gibco; Thermo Fisher Scientific, Inc.). All cells were incubated at 37˚C under 5% CO_2_.

### Reverse transcription-quantitative PCR (RT-qPCR)

RNA was isolated using the TRIzol reagent (Thermo Fisher Scientific, Inc.) or the RNeasy Mini Kit (Qiagen China Co., Ltd.) according to the manufacturers’ protocols. First-strand cDNA was generated using ReverTra Ace qPCR RT Master Mix (Toyobo Life Science). qPCR was performed using TB Green Premix Ex Taq (Takara Bio, Inc.) on the ABI7500 or Vii7 system (Thermo Fisher Scientific, Inc.). Relative expression levels were determined by normalizing to those of GAPDH. The thermocycling conditions used in RT-qPCR are as the following: 37℃, 15 min^*6^; 85℃, 5 sec; 4℃^*7^. The primers were listed as the following: Octamer-binding transcription factor 4 (OCT4) forward, 5’-CTCTTTTGACTGGCCTCCCC-3’ and reverse, 5’-GGGTTTCTGCTTTGCATATCTCC-3’; SRY-box transcription factor 2 (SOX2) forward, 5’-CATGAAGGAGCACCCGGATT-3’ and reverse, 5’-TAACTGTCCATGCGCTGGTT-3’; cell division cycle 20 (CDC20) forward, 5’-ATGGACGACATTTGGCCAGT-3’ and reverse, 5’-CCATGCTACGGCCTTGACAG-3’; Nanog forward, 5’-AATGGTGTGACGCAGGGATG-3’ and reverse, 5’-ACTGTTCCAGGCCTGATTGT-3’ and centromere protein F (CENPF) forward, 5’-CGTCCCCGAGAGCAAGTTTA-3’ and reverse, 5’-GTAGGCAGCCCTTCTTTCCA-3’.

### Construction of stable eIF6-overexpressing or knockout cells

The transfection of LUAD cells with eIF6-overexpressing lentiviral vectors and control vectors were performed using the Lipofectamine 3000 reagent (cat. no. L3000015; Invitrogen; Thermo Fisher Scientific, Inc.) according to the manufacturer’s protocol. Western blotting and RT-qPCR were utilized to assess the expression of eIF6 proteins in transfected cells. For *CRISPR/Cas9*-mediated eIF6-knockout (eIF6-KO), guide oligos targeting eIF6 were cloned into the pX459 plasmid. LUAD cells (A549 and H1299) were then plated and transfected with these pX459 constructs overnight. After 24 h transfection, 1 µg/ml puromycin was used to screen the cells for 3 days. The remaining viable cells were then seeded into the 96-well plates to obtain a monoclonal cell line. eIF6-knock out cell clones were screened by western blotting and validated by Sanger sequencing. Sequences of the gene-specific eIF6-sgRNAs are listed as follows: eIF6-sgRNA1 forward, 5’-CACCGGGCGATAGACGCGTGCACCA-3’ and reverse, 5’-AAACTGGTGCACGCGTCTATCGCCC-3’ and eIF6-sgRNA2 forward, 5’-CACCGCGGTGGTATTGTTGGGTACC-3’ and reverse, 5’-AAACGGTACCCAACAATACCACCGC-3’.

### Cell counting kit-8 (CCK-8), colony formation assay and migration assay

Cell proliferation was measured using a Cell Counting Kit-8 (CCK-8; Dojindo Molecular Technologies, Inc.). Control and transfected LUAD cells at equal density (1,000 cells/well) were first seeded into 96-well plates and cultured at 37˚C with 5% CO_2_, before 10 µl CCK-8 reagent was added to each well 0, 1, 2, 3, 4 and 5 days after plating. The plate was then incubated for an additional 2 h at 37˚C before optical density in each well was measured at 450 nm using an automatic enzyme labeling (Molecular Devices; Thermo Fisher Scientific, Inc.). Each experiment was independently repeated three times in triplicate.

For the colony formation assay, after eIF6 knockout, 500 LUAD cells were seeded into a six-well culture plate and incubated at 37˚C with 5% CO_2_. The colonies became visible at 10 days, which were fixed with 4% paraformaldehyde and stained with crystal violet. The number of colonies, which were defined as those that consisted of ≥ 50 cells, were then counted.

For the migration or invasion assays, a total of 1 × 10^4^ cells were suspended in DMEM medium without FBS before being seeded into the upper chambers of uncoated Transwell chambers. DMEM medium with 20% FBS and conditioned medium (CM) were then placed into the lower chamber. In total, 36 h after seeding, the non-invasive cells that remained on the upper chambers were removed using a cotton swab, whereas cells on the lower surface of the membranes were fixed with 4% paraformaldehyde, stained with crystal violet and imaged at x200 magnification. The data are presented as the means ± standard deviation from three independent experiments.

For the functional rescue assays, we utilized the eIF6-KO NCI-H1299 cells to perform the colony formation and Transwell assays. Meanwhile, we transfected the eIF6 plasmids to the eIF6-KO cells to generate eIF6-OE cell lines and repeat the rescue assays.

### Sphere formation assays

 The A549 and H1299 cells were harvested and re-suspended in serum-free medium (as previously mentioned in tumor specimens and cell culture). After cell counting, 200 cells/well in 200 µl serum-free medium were then added into a 96 well plate, where each group took up 10 wells. The medium was changed every 2 days. Imaging of five randomly selected regions was performed in each group using a fluorescence microscope (Leica Microsystems GmbH). The % sphere formation was calculated as the number of sphere/200.

### Western blot analysis

Total proteins were extracted from tissues or cells using RIPA buffer containing a protease inhibitor cocktail. The protein concentration was determined using a BCA Protein Assay kit (Beyotime Institute of Biotechnology). Proteins were separated by 10–12% SDS-PAGE and transferred onto PVDF membranes (MilliporeSigma). PVDF membranes were then blocked with 5% non-fat milk in 0.1% Tween 20-TBS (TBST) for 1 h at room temperature before being incubated with the primary antibodies overnight at 4˚C. After washing three times for 10 min each in TBST, membranes were incubated with second antibodies for 1 h at room temperature. The membranes were then probed using the ECL Western Blotting Substrate (Beyotime Institute of Biotechnology) and the chemiluminescence imaging system. β-actin was used as the internal control. The antibodies in the present study were anti-eIF6 (cat. no. ab124839; Abcam, 1:5000) and anti-β-actin (cat. no. ab8226; Abcam; 1:5000). The secondary antibody is anti-Mouse IgG (cat. no. ab205719; Abcam, 1:10000).

### Bioinformatic analysis

The gene expression data for LUAD samples and adjacent normal tissues were downloaded from The Cancer Genome Atlas (TCGA) database (https://www.cancer.gov/tcga) and GTEx-Lung dataset. In addition, the eIF6 expression profiles GSE68465 and GSE31210 were obtained from the Gene Expression Omnibus (GEO) database (https://www.ncbi.nlm.nih.gov/geo). All bioinformatics data were analyzed using R software, with fold change ≥ 2 and P ≤ 0.05 considered to indicate a statistically significant difference. The sets of genes that were found to encode proteins that can interact with eIF6 were downloaded from the UniHI website (https://www.unihi.org) and then entered into the Metascape (https://www.metascape.org) online tool to perform pathway enrichment analysis. The data for genes associated with the biological function of eIF6 in liver cancer were downloaded from the R2: Genomics Analysis and Visualization Platform (https://hgserver2.amc.nl/cgi-bin/r2/main.cgi) to analyze the correlation of eIF6 with these genes. The Kaplan-Meier (K-M) Plotter website (https://kmplot.com/analysis/) was used to analyze overall survival (OS) in patients with HCC. The K-M plots and their log rank *P*-values were obtained online. The clinical information of patients with LUAD was summarized in the Table SI. The median value of eIF6 expression in the LUAD samples was 79.99, which was summarized in Table SII.

### Generation of lung cancer xenografts in mice

BALB/c male nude mice were purchased from Shanghai SLAC Laboratory Animal Co., Ltd. All mice were maintained in the mouse facilities. In total, 5 × 10^6^ NCI-H1299 cells (parental and eIF6-KO) were suspended in 100 µl 1X PBS and injected into the flanks of the nude mice. After 7 weeks, all mice were sacrificed. During this time, tumor volumes were recorded every 7 days. The subcataneous tumors were recorded by the ruler every 7 days and the tumor volumes were calculated as the formular: Tumor size was measured using rulers to collect maximal tumor length and width. Tumor volume was estimated with the following formula: V = (L × W2)/2, L represented the length and W represented the width. There are no obvious tumor ulcer. For the establishment of orthotopic lung cancer model, luciferase-tagged A549 cells were injected through the pleura (1.5 × 10^6^ cells in media with Matrigel, 1:1 ratio in volume) into the 7-week-old nude mice. Subsequently, the in situ tumor signals were detected and compared using the in vivo Imaging System (IVIS). All experimental experiments were reviewed and approved by the Ethics Review Committee for Animal Experimentation of Ruijin hospital (Shanghai, China). According to the rules of humanism and ethical requirements, the volumes of subcatenous tumors were less than 1200 mm^3^ and the lung photon flux signals were less than 8*10^6^ in mice. We complied with the ethical guidelines from the USA institutions. Last of all, the percentage of the chamber volume is 70% chamber volume replaced per minute (December, 2021), according to AVMA GUIDELINE for the euthanasia of animals:2018 edition. The death certification is bilateral thorax cut opening. We have provided the approval number with B-2021-018 granted in this study.

### Statistical analysis

All statistical analyses were evaluated using SPSS 22.0 software (IBM Corp.). Quantitative data from three independent experiments are indicated as the means ± standard deviation. One-way ANOVA or two-tailed un-paired Student’s t-test were performed for the comparisons among groups. χ^2^ tests were used to analyze the association between eIF6 expression and the clinicopathological characteristics. The post hoc test used after ANOVA is the Tukey HSD method. The K-M method was performed for survival analysis. Spearman’s correlation coefficient was used to examine the linear relationship between eIF6 expression and that of target genes in LUAD tissues. The *P* < 0.05 was considered to indicate a statistically significant difference.

## Results

### Identification of eIF6 as a hazard factor that expresses highly in LUAD tumor samples

**Fig. 1:**
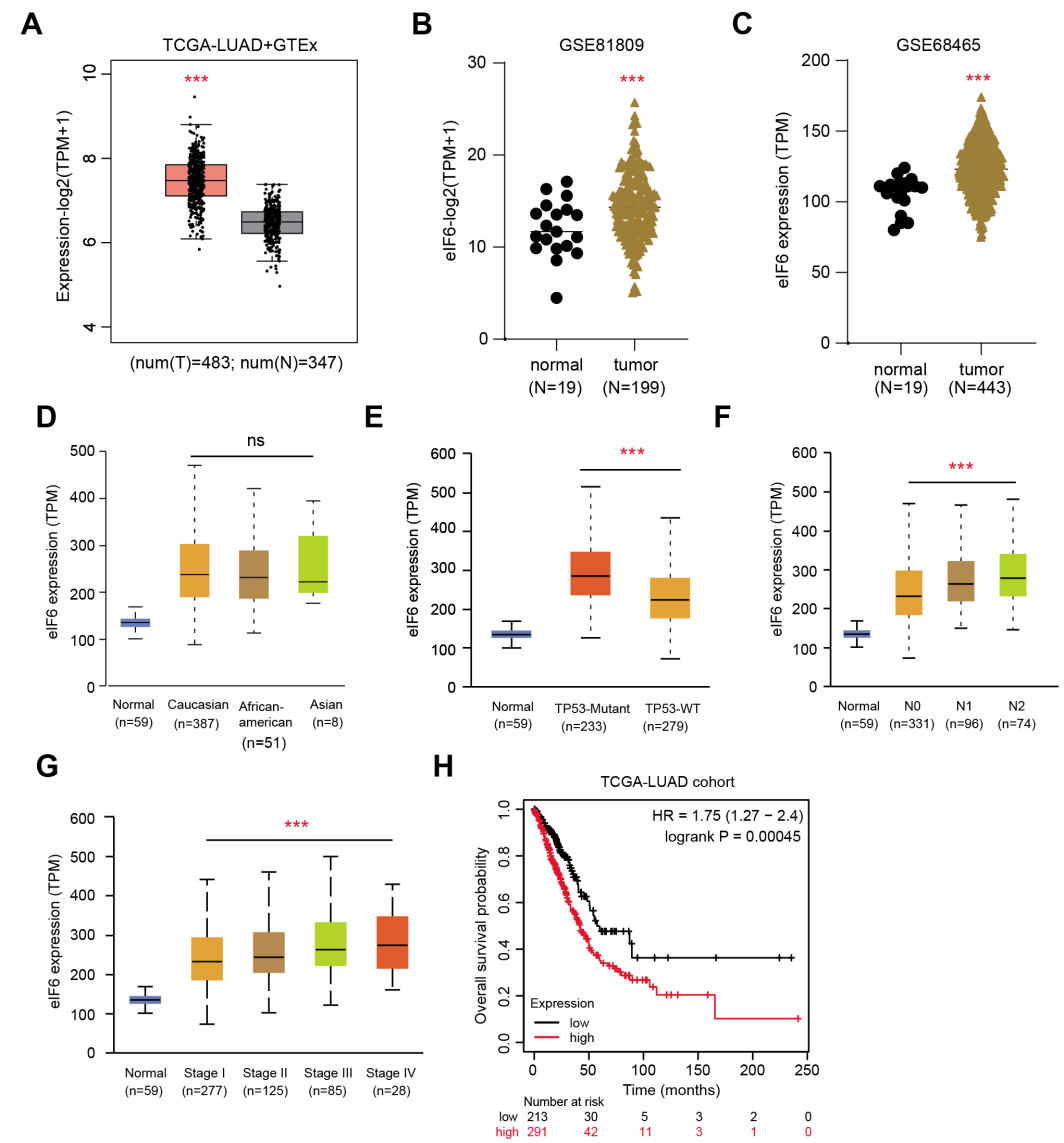
Identification of eIF6 as a prognostic factor in LUAD based on bioinformatic analysis. (A) Boxplot of the TCGA-LUAD dataset showing differential eIF6 mRNA expression in normal and tumor samples. The level eIF6 mRNA expression is higher in tumor samples compared with that in normal tissues in both the (B) GSE81809 (normal, n = 19; tumor, n = 199) and (B) GSE68465 (normal, n = 19; tumor, n = 443) GEO datasets. (D) No significant difference in eIF6 mRNA expression could be observed after stratification by ethnicity. Higher eIF6 mRNA levels were significantly associated with the (E) TP53-mutation genotype, (F) lymphatic metastasis and (G) pathological stages. (H) Kaplan-Meier analysis suggested that patients with higher eIF6 expression levels had shorter overall survival compared with those with lower eIF6 expression levels, according to log-rank test (TCGA-LUAD cohort; n = 504; P = 0.00045). ^***^P < 0.001. eIF6, eukaryotic translation initiation factor 6; LUAD, lung adenocarcinoma; TCGA, The Cancer Genome Atlas; GEO, Gene Expression Omnibus

The expression matrix from the TCGA-LUAD cohort was first downloaded and found that eIF6 expression was significantly higher in the tumor samples compared with that in the normal tissues according to the differential analysis (Fig. 1 A). In addition, the expression levels of eIF6 were observed to be higher in tumor samples compared with those in normal lung tissues via another GEO datasets, including GSE81809 (n = 199) and GSE68465 (n = 443; Fig. 1B and C). It was also found that higher eIF6 expression was associated positively with more advanced TNM and pathological stages, suggesting that eIF6 is a hazard factor (Fig. 1D-G). Subsequently, the LUAD samples were divided into the eIF6-high and eIF6-low groups to conduct the Kaplan-Meier analysis, where patients with higher eIF6 expression levels were associated with worse survival prognosis compared with those with lower expression levels of eIF6 (n = 494; Fig. 1 H). Taken together, these results suggest that eIF6 is a hazard factor in LUAD such that higher expression levels of eIF6 are associated with more unfavorable clinicopathological parameters.

### eIF6 can be an effective biomarker for predicting poor prognosis

**Fig. 2:**
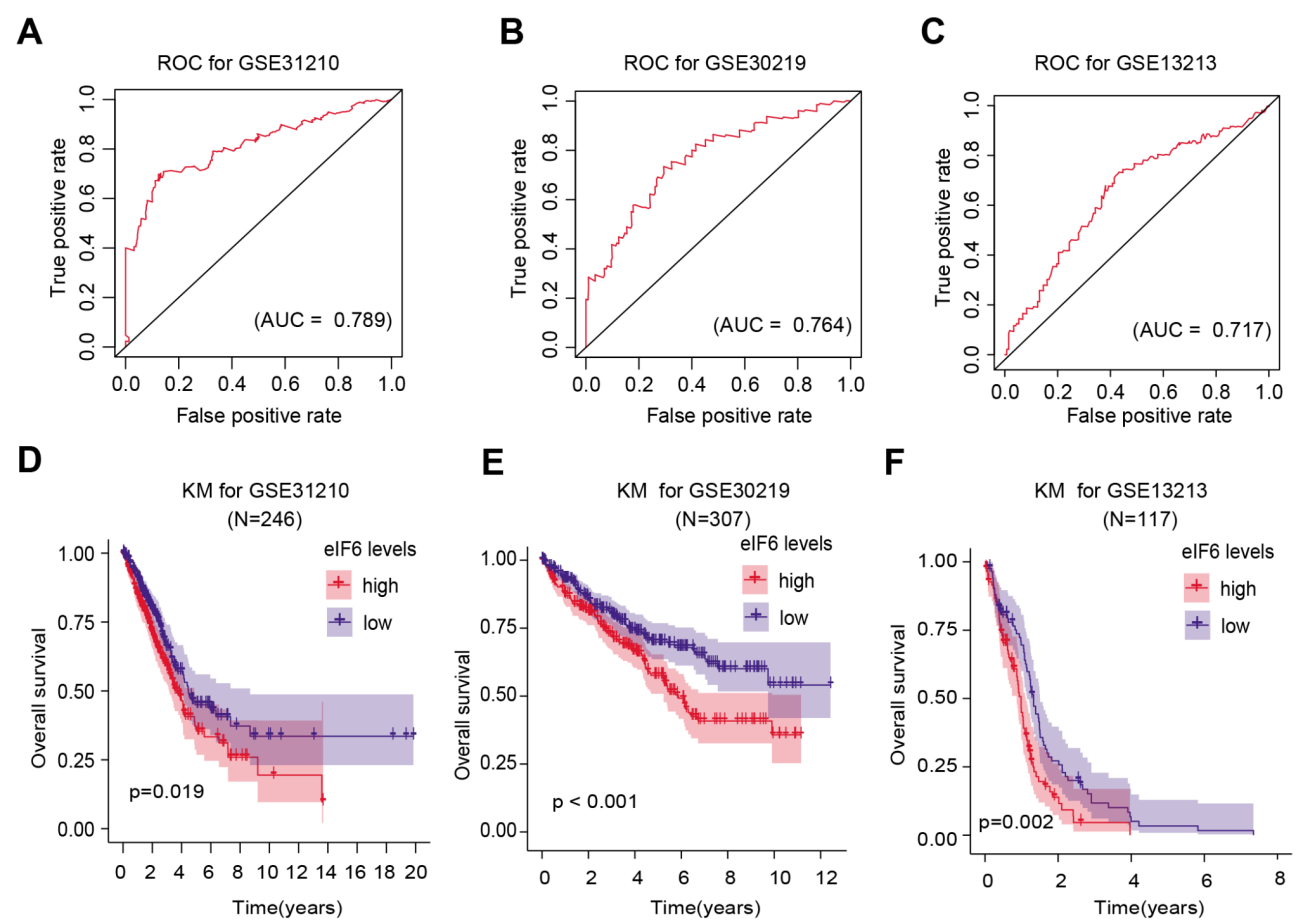
Bioinformatic analysis reveals the superior predictive values of high eIF6 expression levels. (A) ROC curve for eIF6 expression in normal and LUAD tissues from the GSE31210 dataset. AUC = 0.789. (B) ROC curve for eIF6 expression in normal and LUAD tissues from the GSE30219 dataset. AUC = 0.764. (C) ROC curve for eIF6 expression in normal and LUAD tissues from the GSE13213 dataset. AUC = 0.717. (D) Kaplan-Meier analysis indicated that patients with higher expression eIF6 levels were associated with poorer OS outcomes compared with those in patients with lower expression eIF6 levels according to the GSE31210 dataset (n = 246). Log-rank test, P = 0.019. (E) Kaplan-Meier analysis indicated that patients with higher eIF6 expression levels were associated with poorer OS outcomes compared with those in patients with lower eIF6 levels according to the GSE30219 dataset (n = 307). Log-rank test, P = 0.019. (F) Kaplan-Meier analysis indicated that patients with higher eIF6 expression levels were associated with poorer OS outcomes compared with those in patients with lower eIF6 levels according to the GSE13213 dataset (n = 117). Log-rank test, P = 0.019. ^***^P < 0.001. eIF6, eukaryotic translation initiation factor 6; LUAD, lung adenocarcinoma;ROC, receiver operating characteristic; AUC, area under the curve; OS, overall survival

Receiver Operating Characteristic (ROC) curves were then used to evaluate the clinical significance of eIF6 expression in LUAD. The area under the ROC curve (AUC) from GSE31210 was calculated to be 0.789, suggesting a well diagnostic power (Fig. 2 A). In addition, the AUC values from patients with LUAD in the GSE30219 and GSE13213 datasets were also calculated, yielding 0.764 and 0.717, respectively (Fig. 2B and C). Kaplan-Meier analysis in other independent LUAD datasets was also conducted, which verified that patients with higher eIF6 expression levels were associated with worse survival outcomes compared with those with lower eIF6 expression levels (Fig. 2D and E).

### eIF6 promotes LUAD cell proliferation in vitro and in vivo

**Fig. 3:**
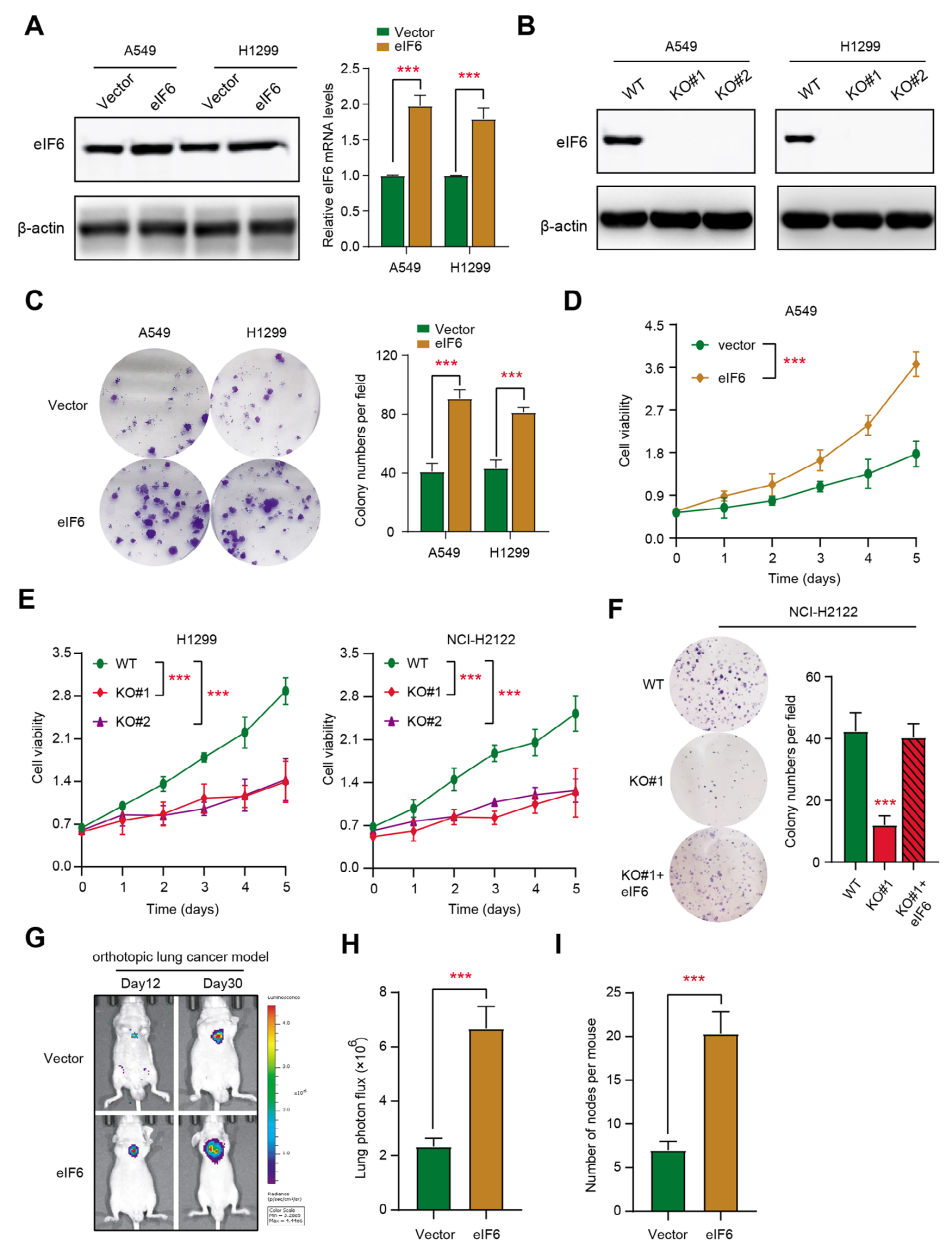
eIF overexpression promotes LUAD cell proliferation in vitro and growth in vivo. (A) Western blotting assay and reverse transcription-quantification revealed that eIF6 was overexpressed in A549 and NCI-H1299 cells. (B) *CRISPR/Cas9* technology was utilized to knock out eIF6 in LUAD cell lines A549 and NCI-H1299. (C) eIF6 overexpression was able to enhance the colony formation ability of LUAD cells, which was quantified. (D) Cell Counting Kit-8 assay revealed that eIF6 could elevate the in vitro cell proliferation of A549 cells. (E) eIF6 knockout notably suppressed the proliferation of H1299 and NCI-H2122 cells. (F) eIF6 knockout notably decreased the colony formation capabilities, which was rescued by the overexpression of eIF6. (G) Representative bioluminescence imaging images of the orthotopic lung cancer models in the control and eIF6-OE groups, (H) which was quantified. (I) Quantification of the tumor nodes in the lung. ^***^P < 0.001. eIF6, eukaryotic translation initiation factor 6; LUAD, lung adenocarcinoma; OE, overexpression

To investigate the underlying biological function of eIF6 in LUAD cells, stable eIF6-knockout A549 and H1299 cell lines were established through *CRISPR/Cas9* technology. In addition, stable eIF6-overexpressing A549 and H1299 cells were constructed using lentiviral infection. The overexpression or knockout efficiency of eIF6 was then assessed using western blotting in three independent cell lines (Fig. 3 A and B). Colony formation assay data suggested that the number of colonies derived from the eIF6-overexpressing cells (A549 and H1299) was significantly increased compared with that of control cells, which was indicated by the quantitative analysis (Fig. 3 C). CCK-8 assays also revealed that eIF6 overexpression can potentiate the cell proliferation of A549 cells compared with that in cells transfected with the negative control vector (Fig. 3D). By contrast, eIF6 knockout following CRISPR notably decreased cell proliferation compared with that in the control group, as indicated by results from CCK-8 assays (Fig. 3E). eIF6 knockout also markedly decreased the number of colonies formed by A549 cells, whereas eIF6 overexpression restored this cell proliferation (Fig. 3 F). Rescue assays were conducted in H1299 cells. A549-derived orthotopic lung tumor models were next established, which found that eIF6 overexpression could markedly drive the progression of tumor cells in vivo compared with that in the vector control groups, as indicated by the bioluminescence imaging signals and number of tumor nodes recorded per mouse (Fig. 3G-I).

**Fig. 4:**
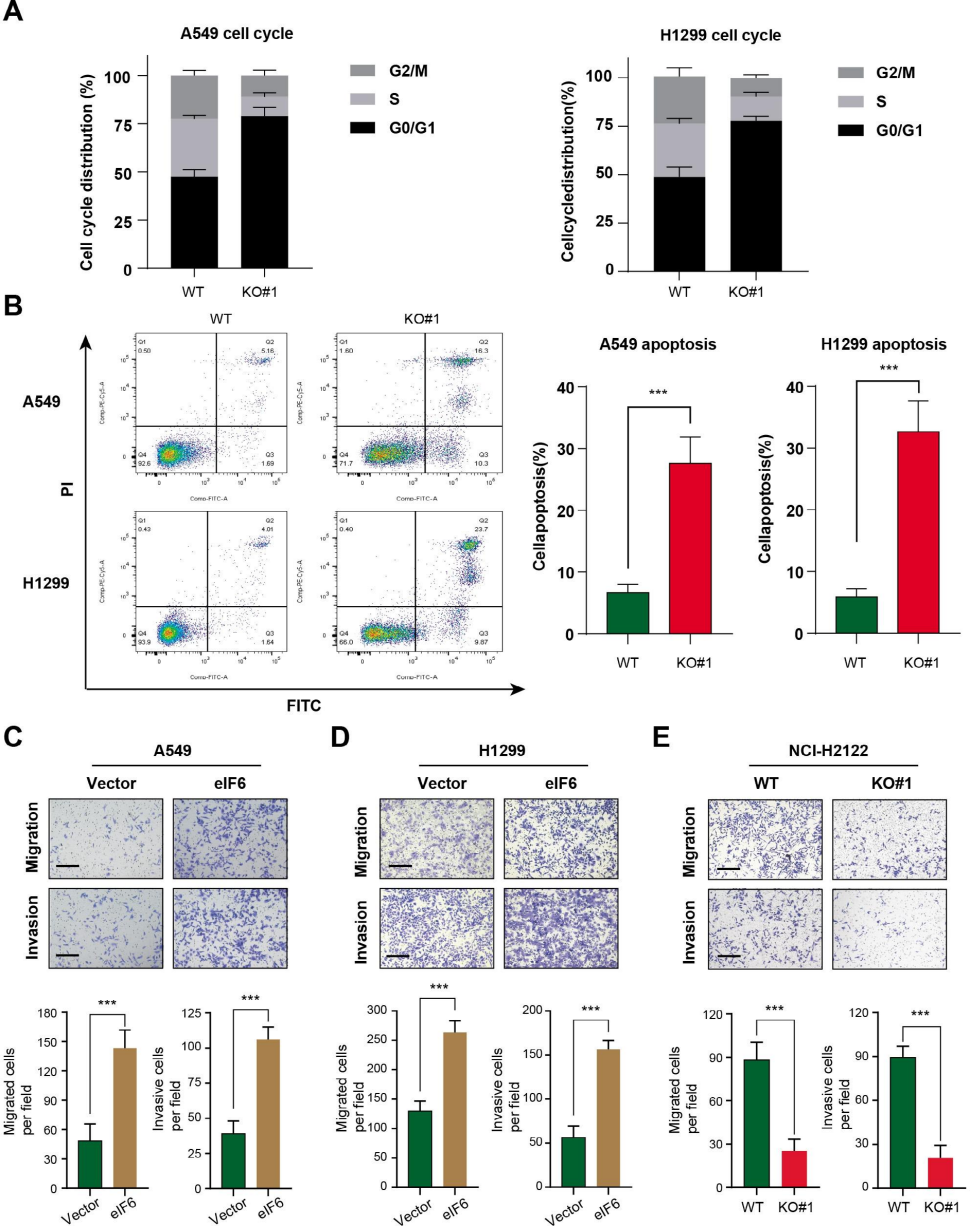
Knockout of eIF6 expression induces cell cycle arrest and apoptosis whereas eIF6 overexpression enhances cell migration. (A) Cell cycle assay of control cells and eIF6-KO cells was conducted by flow cytometry, where cell cycle distribution was quantified and compared from three independent assays. (B) Cell apoptosis assay was performed by flow cytometry, where the cell apoptosis rate was calculated and compared in the control and eIF6-KO cells. eIF6 overexpression enhanced (C) cell migration and (D) invasion, (E) whilst eIF6 KO exerted the opposite results. eIF6, eukaryotic translation initiation factor 6; KO, knockout

Flow cytometry assay was then conducted to detected the effect of eIF6 on apoptosis and the cell cycle progression in A549 and H1299 cells. Consistent with the findings aforementioned [14], eIF6 knockout markedly increased the proportion of LUAD cells in the G_0_/G_1_ phases whilst markedly reducing the proportion of cells in S phase (Fig. 4 A). Knocking out eIF6 expression also increased the total apoptosis rate in A549 and H1299 cells (Fig. 4B). Furthermore, Transwell-Matrigel assays were performed to determine the effects of eIF6 knockout on cell invasion. eIF6 knockout was found to markedly reduce the migratory and invasive capacities of LUAD cells (A549, H1299 and NCI-H2122) in vitro (Fig. 4 C-E). Of note, as eIF6 proteins completely locate in the cell nucleus (https://www.proteinatlas.org/ENSG00000242372-EIF6), we did not need to evalaute the levels of eIF6 expression protein in the two cell compartments.

In conclusion, these results show that eIF6 drives the malignant progression of LUAD in vitro and in vivo.

**Fig. 5:**
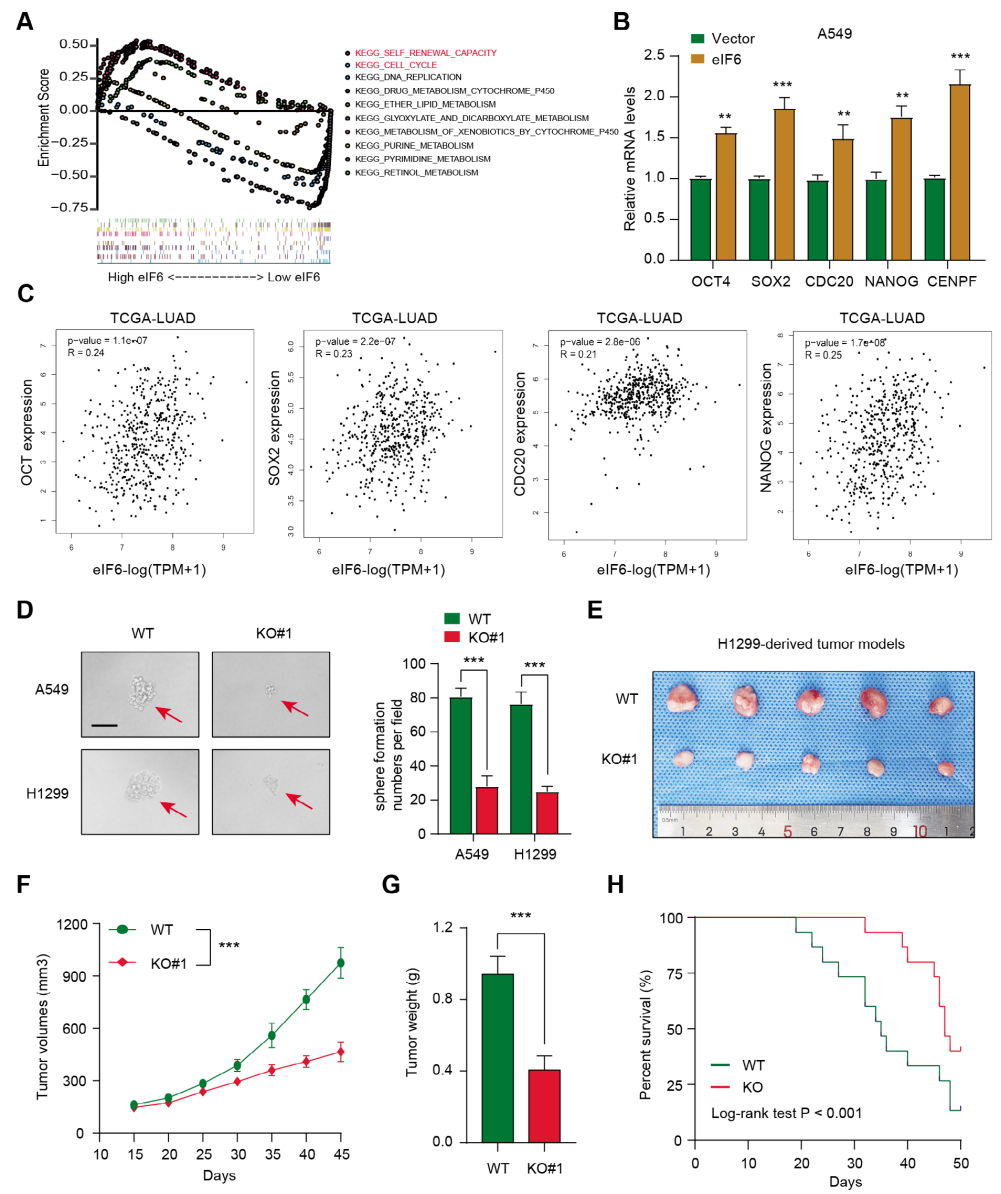
eIF6 is associated with the stemness process such that targeting eIF6 impairs growth in vivo. (A) Gene set enrichment analysis was conducted in eIF6-high and eIF6-low samples. (B) Reverse transcription-quantitative PCR assay was used to measure the mRNA expression of representative stemness-associated genes in Ctrl and eIF6-OE cells. (C) Correlation analysis revealed the positive associations between eIF6 expression and the stemness signature in The Cancer Genome Atlas-Lung adenocarcinoma cohort. (D) Sphere formation assays were conducted in Ctrl and eIF6-OE cells, which were quantified. (E) Representative images showing the subcutaneous xenograft mouse model in Ctrl and eIF6-KO groups. Quantification of (F) tumor volumes and (G) tumor weight in two groups. Tumor volumes were recorded every 7 days. (H) Kaplan-Meier analysis of the survival time of mice in both Ctrl and eIF6-KO groups. ^***^P < 0.001. eIF6, eukaryotic translation initiation factor 6; Ctrl, control; OE, overexpression

*eIF6 correlates with stemness and eIF6 knockout can suppress LUAD growth in vivo.* To elucidate the underlying signaling pathways by which eIF6 mediates the malignant progression of LUAD, LUAD samples were divided again into the eIF6-high and eIF6-low groups before GSEA was conducted between the two groups. It was found that a number of oncogenic crosstalk were enriched in the eIF6-high LUAD samples, including self-renewal capacity, cell cycle, DNA replication and drug-metabolism (Fig. 5 A). Since previous studies have already reported the association between eIF6 and cell cycle progression, the potential relationship between eIF6 expression and the tumor self-renewal features was next focused upon. RT-qPCR was first performed, which found that eIF6 overexpression resulted in the elevation of the expression of representative stemness-associated genes, including OCT4, SOX2, CDC20, Nanog and CENPF, in A549 cells (Fig. 5B). Accordingly, this potential relationship between eIF6 and the stemness signature could also be found in the TCGA-LUAD samples (Fig. 5 C). A reduction in sphere numbers and sizes were observed in eIF6-knockout A549 and H1299 cells compared with those in control cells (Fig. 5D). Subsequently, a subcutaneous xenograft model was established as previously reported [22, 23], which found that eIF6 knockout could markedly delay tumor growth compared with that derived from the control cells, as indicated by the tumor volume and weight measurements (Fig. 5E-G). Kaplan-Meier analysis with log-rank tests suggested that mice bearing tumors derived from eIF6-KO cells were associated with superior prognosis compared with that in mice bearing tumors from control cells (Fig. 5 H). These results suggest that eIF6 can manipulate the tumor self-renewal ability to promote LUAD progression.

**Fig. 6:**
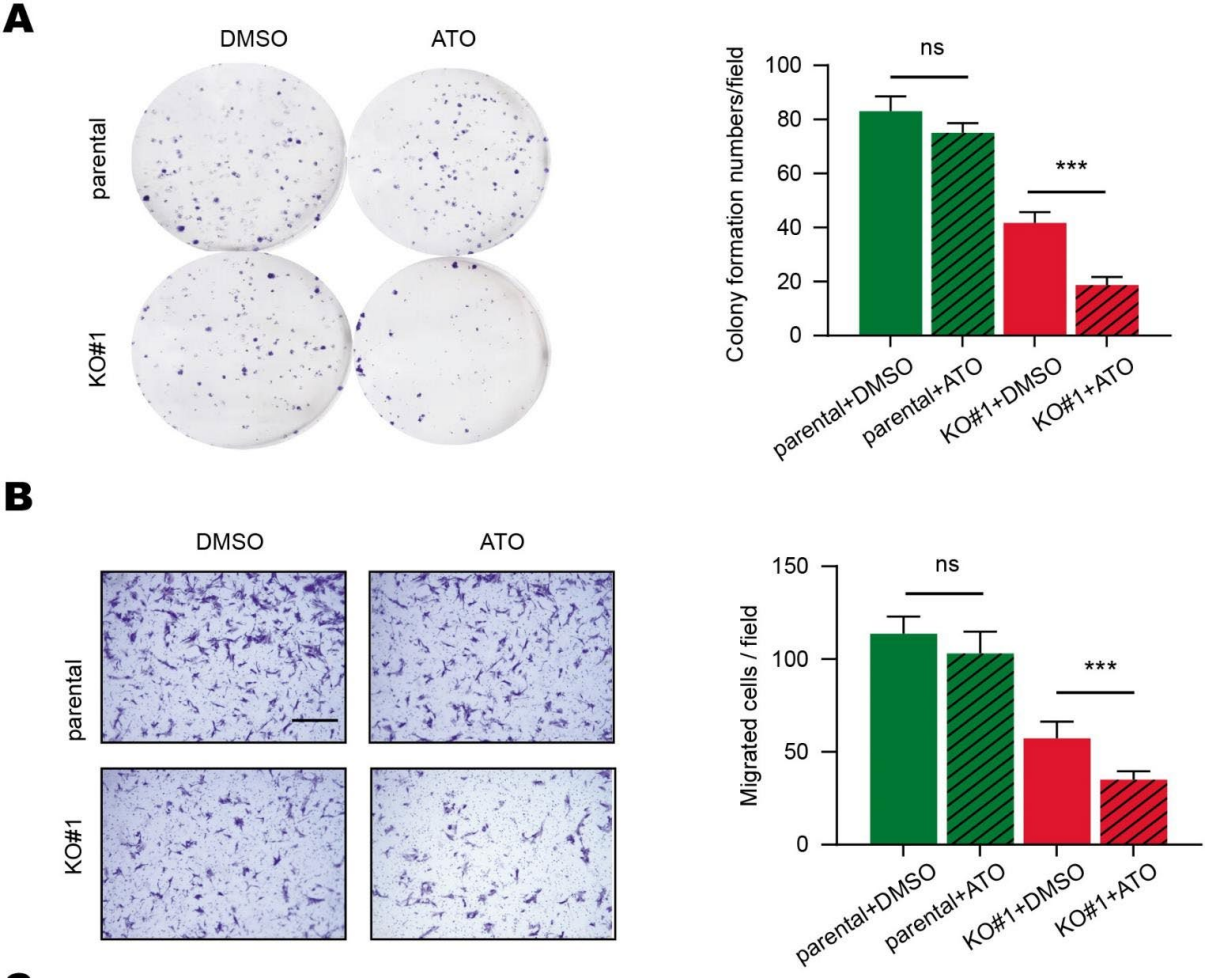
Targeting eIF6 expression has a synergistic effect with ATO treatment. (A) Colony formation assays of A549 cells following ATO treatment in the indicated groups. (B) Migratory abilities of A549 cells after ATO treatment in the indicated groups. Scale bars, 250 μm. ^***^P < 0.001. eIF6, eukaryotic translation initiation factor 6; ATO, arsenic trioxide

The potential association between eIF6 expression and arsenic trioxide (ATO) treatment efficacy was next investigated. eIF6-overexpressing A549 cells were found to be resistant to ATO treatment, which only mildly decreased cell proliferation and migration (Fig. 6 A and B). However, eIF6-KO rendered the A549 cells sensitive to ATO treatment both in vitro (Fig. 6 A-B). Taken together, these data suggest that eIF6 can be exploited as a therapeutic target for LUAD, since it exhibited a synergistic effect with ATO treatment.

## Discussion

Lung adenocarcinoma is the most prevalent subtype among all types of lung cancers. Improvements have been made in the development of treatment strategies of LUAD, including in the fields of immunotherapy, non-invasive surgical resection and radiotherapy [24–26]. However, the overall outcome of patients with LUAD remain pessimistic, with a ~ 20% 5-year OS rate [27]. Over the past decade, the notion of ‘precision medicine’ idea has been widely encouraged for optimizing molecular detection technologies and targeted therapies. Key genes driving LUAD pathogenesis can be exploited for discovering novel therapeutic targets for cancer treatment. EGFR mutations account for the majority of all druggable targets in patients with LUAD. The optimization of EGFR inhibitors has greatly improved the quality of life of patients with metastatic lung cancer. In addition, multi-omics clustering analysis has also previously identified four subgroups according to key driver mutations, geographic location and sex. In particular, proteomic and phospho-proteomic data were used to depict the biological consequence of copy number aberrations, somatic mutations and fusions, in addition to driver events involving KRAS and anaplastic lymphoma kinase [28]. Furthermore, immune profiling also revealed a comprehensive landscape, exemplified by the underlying relationship between serine/threonine kinase 11 and immune-cold behavior, suggesting a potential immunosuppressive role of neutrophil degranulation [29]. Consequently, searching for key drivers would be useful for finding targets to treat LUAD. In the present study, differential expression analysis was performed, which found that eIF6 expression is higher in LUAD tissues compared with that paired normal lung tissues. Correlation analysis also found that higher eIF6 expression levels were positively associated with rik clinicopathological factors, such as the TP53 mutation status, clinical and higher-grade tumor stages. This finding was also made following the analysis of other independent LUAD cohorts in the GSE31210, GSE30219 and GSE13213 datasets. The eIF6 expression levels potentiate the superior predictive efficiency of prognosis in patients with LUAD. This study evaluated the biological roles of eIF6 in lung cancer and found the associations between eIF6 and malignant features of lung cancer. Apart from previous studies, this paper further indicated the functional roles of eIF6 in modulating tumor stemness and identified eIF6 as a therapeutical target in lung cancer.

eIFs have been frequently reported to serve key roles in the translation of proteins that control cell proliferation, apoptosis and malignancy. Since accelerated protein synthesis is one of the hallmarks of cancer, eIFs have been previously observed to be dysregulated, which in turn contributed to more aggressive characteristics. Previous studies have revealed that the overexpression of proteins in the eIFs family is an essential process in a wide variety of cancers. eIF4F has been found to bind to the 5’ cap of mRNAs to regulate the expression levels of IFN-γ-induced programmed death-ligand 1 on cancer cells [30]. Mechanistically, eIF4F is mainly responsible for regulating the translation of the transcription factor STAT1. Formation of the eIF4F complex was proposed to predict and associate with the clinical efficacy of immunotherapy in human melanoma. In addition, eIF4A was demonstrated to enhance T-cell acute lymphoblastic leukemia progression in vivo and is necessary for leukemic carcinogenesis [31]. Transcriptomic data revealed that the eIF4A-dependent and silvestrol-sensitive transcripts included a number of oncogenes, super enhancer-associated transcription factors and epigenetic modifiers. Basic leucine zipper and W2 domains 1 is an adaptor for protein kinase RNA-like endoplasmic reticulum kinase, which in turn facilitates the phosphorylation of eIF2α to promote internal ribosome entry site (IRES)-dependent translation of HIF1α and c-Myc in human pancreatic ductal adenocarcinoma [32]. However, to the best of our knowledge, the potential association between eIF6 and lung cancer remain poorly understood. In the present study, eIF6 was found to promote LUAD cell proliferation and colony formation in vitro, whilst increasing *in-situ* tumor growth. Targeting eIF6 could notably suppress cancer cell proliferation and induce cancer cell apoptosis. In addition, eIF6 overexpression was found to significantly promote cell migration and invasion. These results suggest that eIF6 can not only be a marker for predicting prognosis, but may also be required for lung cancer cell proliferation and growth.

To investigate the downstream pathways that can be regulated by eIF6 in LUAD, bioinformatics analysis based on the transcriptomic data in eIF6-high and eIF-low samples was performed. Higher eIF6 expression levels were found to associate positively with several well-known oncogenic processes, including self-renewal, cell cycle process and DNA replication. Subsequently, eIF6 was detected to increase the expression of representative stemness-associated genes in A549 cells, including OCT4, SOX2, CDC20 and NANOG. This suggests that that targeting eIF6 can suppress cell stemness. Accumulating evidence has indicated that cancer stem-like cells accumulate through the reprogramming of adult stem cells or progenitor cells, which are required for tumor initiation, maintenance and progression [33, 34]. Deepening the understanding into the underlying mechanisms that contribute to the maintenance of stem-like features would lead to the improvement in methods for clinical management. In fact, specifically inhibiting cancer stemness has been proposed to be a useful strategy for improving the overall prognosis of patients with LUAD [35]. Jiang et al. [27] reported that hypoxia-induced factor-1α can maintain the stem cell-like phenotypes to promote the progression of LUAD by simultaneously activating Wnt/β-catenin and Notch signaling, through regulating miR-1275 expression [36]. In addition, Nanog functions as a cancer stemness indicator, which enhances cancer tumorigenesis and stemness [37]. Aberrant Nanog expression has been frequently observed in several cancer types, including LUAD, where it is known to promote epithelial-mesenchymal transition [38]. Previous studies found that Nanog can maintain the stemness features of liver kinase B1-deficient LUAD and prevent gastric differentiation [39]. In the present study, eIF6 was found to elevate the expression of several stemness-associated genes in a panel of LUAD samples tested. Therefore, it could be hypothesized that eIF6 can promote stemness to maintain the self-renewal capacity of LUAD cells and promote tumorigenesis. Therefore, a subcutaneous xenograft tumor model was established, which found that eIF6 KO can suppress tumor growth in vivo to improve the OS time of mice, compared with the tumors derived from the parental cells. These data suggest drug discovery protocols could potentially target eIF6 for the clinical treatment of LUAD.

However, a number of limitations remain associated with the present study. The specific epigenetic regulation mechanism of eIF6 on the stemness-associated genes identified were not studied. The sample size of the LUAD tissues used to detect the expression levels of eIF6 and to evaluate the prognostic significance of eIF6 needed to be increased. Additional tumor models would need to be applied for assessing the clinical efficacy of eIF6 inhibition in LUAD treatment, including patient-derived xenografts, patient-derived organoid and orthotopic human lung cancer models. Meanwhile, we did not assess the levels of eIF6 expression proteins in the two cell compartments, which could be further clarified in the future studies. Next, as is well documented, p53 mutations represent the genetic hallmark of most malignancies. We just found the positive associations between high eIF6 levels and p53 mutation, implicating that eIF6 is a hazard factor. The potential mechanisms should be performed and investigated in large experimental assays in the following researches. Furthermore, we have conducted the functional enrichment analysis and find that eIF6 was mainly associated with self-renewal process, cell cycle, DNA replication and other crosstalk. In the following researches, we would investigate the reasons that contribute to the high eIF6 levels in LUAD cells, such as WNT or STAT signaling. Last of all, the in-depth mechanisms that how eIF6 regulate these stemness-associated targets remain to be unclear, which need to be further explored.

Taken together, the present study analyzed the public datasets and found that eIF6 is a potential prognostic factor in LUAD. Higher eIF6 expression can promote tumor progression in vitro and in vivo, including increasing cell proliferation and migration. In addition, an underlying association between eIF6 and the self-renewal ability of LUAD cells was uncovered. Therefore, targeting eIF6 may create a novel therapeutic vulnerability for LUAD treatment, which may synergize with ATO treatment.

## Electronic supplementary material

Below is the link to the electronic supplementary material.

Supplementary Material 1

Supplementary Material 2

Supplementary Material 3

## Data Availability

The data used or analyzed during this study are included in this article and available from the corresponding author upon reasonable request.

## Abbreviations

TCGA: The Cancer Genome Atlas
GEO: Gene Expression Omnibus
eIF6: Eukaryotic initiation factor 6
LUAD: lung adenocarcinoma
CCK-8: Cell Counting Kit-8
EGFR: epidermal growth factor receptor
ROC: receiver operating characteristic
