# Supplementary figures and images for: Identification of eIF6 as a prognostic factor that drives tumor progression and predicts arsenic trioxide efficacy in lung adenocarcinoma
Source: Mol Biol Rep. 2022 Nov 26;50(2):1167–80. doi: 10.1007/s11033-022-07917-w (PMC9889454; doi:10.1007/s11033-022-07917-w)

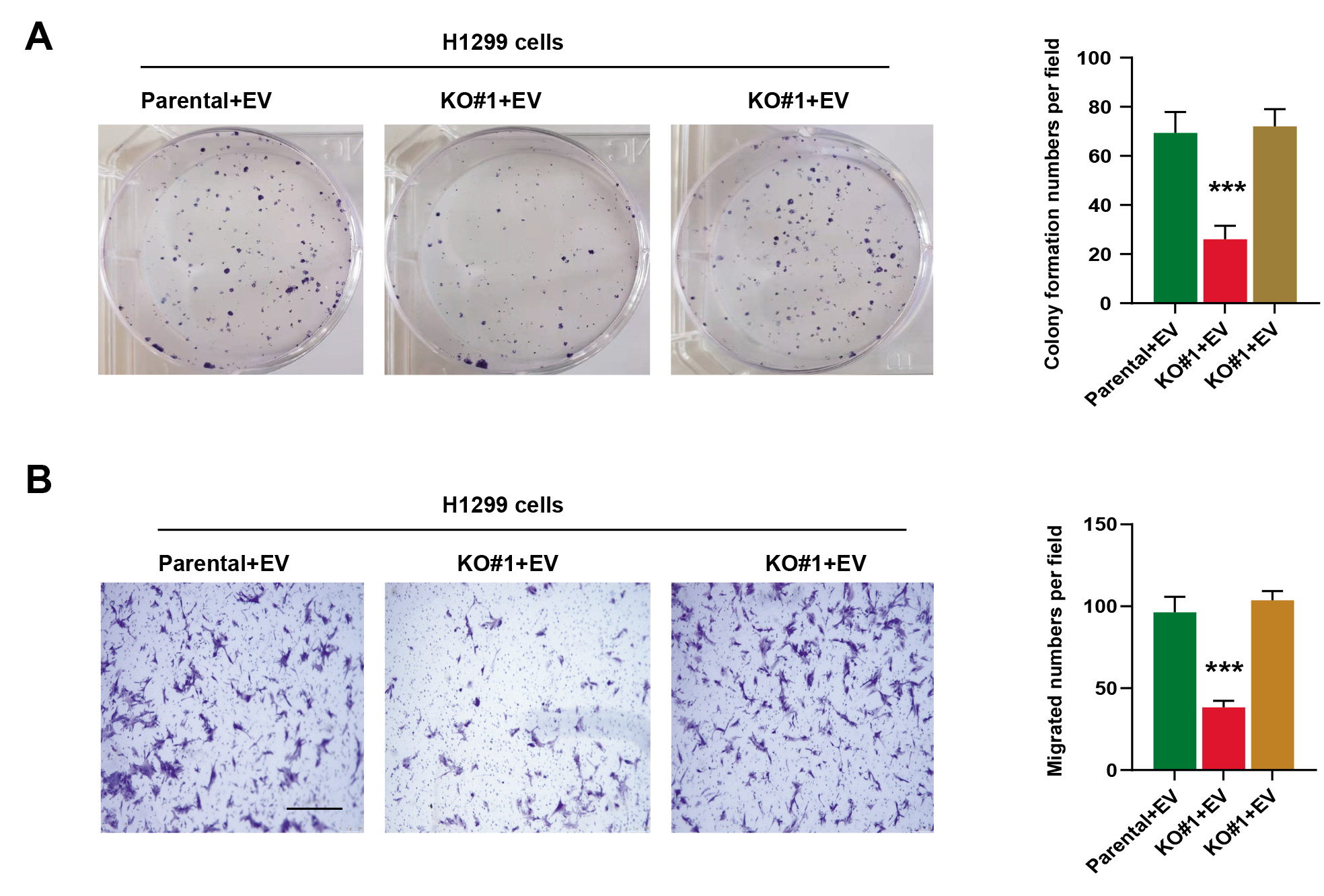

Supplement: Supplementary file 1 — Supplementary Material 1 [file 11033_2022_7917_MOESM1_ESM.jpg]
